# Molecular Mechanism Involved in the Pathogenesis of Early-Onset Epileptic Encephalopathy

**DOI:** 10.3389/fnmol.2019.00118

**Published:** 2019-05-15

**Authors:** Giovanna Vitaliti, Piero Pavone, Silvia Marino, Marco Andrea Nicola Saporito, Giovanni Corsello, Raffaele Falsaperla

**Affiliations:** ^1^Unit of Pediatrics and Pediatric Emergency, University Hospital “Policlinico-Vittorio Emanuele”, Catania, Italy; ^2^Neonatal Intensive Care Unit, Santo Bambino Hospital of Catania, Policlinico-Vittorio Emanuele University Hospital, University of Catania, Catania, Italy; ^3^Department of Maternal and Child Health, University of Palermo, Palermo, Italy

**Keywords:** biological markers, epileptic encephalopathy, inflammation, pathogenic mechanisms, childhood

## Abstract

Recent studies have shown that neurologic inflammation may both precipitate and sustain seizures, suggesting that inflammation may be involved not only in epileptogenesis but also in determining the drug-resistant profile. Extensive literature data during these last years have identified a number of inflammatory markers involved in these processes of “neuroimmunoinflammation” in epilepsy, with key roles for pro-inflammatory cytokines such as: IL-6, IL-17 and IL-17 Receptor (IL-17R) axis, Tumor-Necrosis-Factor Alpha (TNF-α) and Transforming-Growth-Factor Beta (TGF-β), all responsible for the induction of processes of blood-brain barrier (BBB) disruption and inflammation of the Central Nervous System (CNS) itself. Nevertheless, many of these inflammatory biomarkers have also been implicated in the pathophysiologic process of other neurological diseases. Future studies will be needed to identify the disease-specific biomarkers in order to distinguish epilepsies from other neurological diseases, as well as recognize different epileptic semiology. In this context, biological markers of BBB disruption, as well as those reflecting its integrity, can be useful tools to determine the pathological process of a variety of neurological diseases. However; how these molecules may help in the diagnosis and prognostication of epileptic disorders remains yet to be determined. Herein, authors present an extensive literature review on the involvement of both, systemic and neuronal immune systems, in the early onset of epileptic encephalopathy.

## Introduction

Epilepsy affects about 70 millions of people worldwide. A third of epileptic patients never achieve freedom from seizures, even when they follow a therapy with first-choice antiepileptic drugs. These cases are included into the group of drug-resistant epilepsies (DRE), defined as epilepsies not responding to two or more anticonvulsant drugs of first choice for that specific epileptic semiology (van Campen et al., 2014; Wilson and Task Force Members, 2015; Walker et al., 2016). Moreover, in clinical practice, administration of anticonvulsants with different mechanisms of action does not often mitigate drug-resistance (Wilson and Task Force Members, 2015).

Recent studies have shown that neurologic inflammation may both precipitate and sustain seizures, suggesting that inflammation may be involved not only in epileptogenesis but also in the onset of the drug-resistant profile (Wilson and Task Force Members, 2015). This hypothesis has been confirmed by a good response to steroids in epileptic patients and also in children with focal seizures, not traditionally believed to be inflammatory in origin (Marchi et al., 2011; Wilson and Task Force Members, 2015).

As far as the inflammatory cascade in epileptic patients is concerned, studies on individuals with focal drug-resistant epilepsy have shown a pro-inflammatory pattern with an altered interleukin-1beta/interleukin-1 receptor antagonist (IL-1β/IL-1Ra) ratio (Hulkkonen et al., 2004). In rodents, pharmacological blockade of IL-1β biosynthesis significantly decreases seizures frequency by targeting the specific IL-1-converting enzyme responsible for its bioactive form (Maroso et al., 2011a). Moreover, literature data have demonstrated that a “pro-inflammatory cytokine profile” with elevated IL-6 and low IL-1β/IL-1Ra ratio in peripheral blood can identify the patients in whom persistent, unresolved inflammation leads to neuromodulation and alterations in neuronal excitability (Maroso et al., 2011a; Wilson and Task Force Members, 2015). These findings are further supported by human studies looking at the levels of pro-inflammatory cytokines in peripheral blood of subjects with epilepsy (Lehtimäki et al., 2010; Mao et al., 2013). Elevated levels of IL-1β were found in serum and in CSF in subjects with increased risk of developing epilepsy following brain injury (Diamond et al., 2014). Furthermore, the patients with C to T genotype rs1143634 showed lower serum IL-1β levels and higher IL-1β CSF/serum ratios, seemingly associated with both, seizure frequency and the probability of developing subsequent epilepsy (Wathen and Janigro, 2014).

Experimental models of neuroinflammation have suggested that damages in the neuronal tissue and the onset of spontaneous recurrent seizures are modulated *via* complex interactions between adaptive and innate immunity (Vezzani et al., 2008). It has also been suggested that inflammation can occur as a result of ongoing seizures. The authors showed that in animal models seizure activity can induce neuroinflammation and recurrent seizures maintain chronic inflammation (Vezzani et al., 2008).

Overt the last years, research has identified a number of inflammatory markers involved in the process of *“neuroimmunoinflammation”* in epilepsy, further exploring a role for pro-inflammatory cytokines such as: IL-6 (Vezzani et al., 1999; De Simoni et al., 2000), IL-17 and IL-17 Receptor (IL-17R) axis (He et al., 2013), Tumor-Necrosis-Factor Alpha (TNF-α) and Transforming-Growth-Factor Beta (TGF-β), thought to be responsible for the early process of blood-brain barrier (BBB) disruption and a subsequent inflammation of the Central Nervous System (CNS; Viviani et al., 2003; Dubé et al., 2005; Heida and Pittman, 2005; Balosso et al., 2008; Roberts and Goralski, 2008; Maroso et al., 2010; Riazi et al., 2010; Andersson and Tracey, 2011; Vitaliti et al., 2014).

There is growing evidence from experimental models that inflammatory molecules participate in the initiation and continuation of seizures. Furthermore, markers of inflammation have been found in human epileptogenic tissue. These findings suggest that there might be specific inflammation-related pathways that can be targeted to control seizures refractory to conventional anticonvulsant drugs (Roberts and Goralski, 2008).

Herein, authors present an extensive review of the literature on the involvement of both systemic and neuronal immune system in the early onset of epileptic encephalopathy.

## Early Epileptic Encephalopathy and the “Two-Hit Hypothesis” for Subsequent Development of Epilepsy

Neonatal seizures may be associated with permanent dysfunction of synaptic communication, and lead to aberrant hyperexcitable or hypoexcitable circuits. At the molecular level, neonatal seizures modify neurotransmission (Baram, 2012). As a result neonates who experienced seizures are at risk of developing epilepsy at an early age (Ellenberg et al., 1984); the incidence of epilepsy within the first year of life is 73.3% (Pisani et al., 2012). This clinical phenomenon may be interpreted as expression of epileptogenesis. Studies using animal models showed that after an initial insult, there is a change in the regulation of ion channels and receptors, as well as an induction of immediate early genes. The subsequent subacute phase is characterized by neuronal death, microglial activation, neurotrophic factors expression (with the following modulation of synaptic plasticity and maturational onset of potassium chloride co-transporter 2-KCC2 expression) and change in transcriptional. Final chronic stage is characterized by sprouting, neurogenesis, and gliosis, although differences are observed depending on the subjects’ age and experimental model (Rakhade and Jensen, 2009).

In some cases, the initial brain damage requires a second hit to develop spontaneous recurrent seizures (Spagnoli et al., 2015). This event has been called “The Two-Hit Hypothesis” and it suggests that seizures in the immature brain result in changes that make the mature brain more prone to seizure-induced injury in adulthood (Hoffmann et al., 2004). In fact, in murine two-hit models of fluorothyl-induced (Schmid et al., 1999) and kainate-induced (Koh et al., 1999) neonatal seizures, the onset of a neonatal insult has been associated with an increased susceptibility to seizure-induced injury later in life (Schmid et al., 1999), both in adolescence (Schmid et al., 1999) and in adulthood even in the absence of cell loss (Koh et al., 1999; Schmid et al., 1999). Furthermore, in postnatal day 15 rats the occurrence of a status epilepticus has been reported to increase sensitivity to later-life insults (Koh et al., 1999).

In literature, to our knowledge, there is only one study showing the association between the onset of neonatal seizures, febrile seizures, and the development of subsequent epilepsy in preterm babies (Herrgård et al., 2006). These authors found that over five children developing epilepsy, two had also fever-induced seizures (Herrgård et al., 2006). Nevertheless, the authors did not provide the age at onset of either febrile seizures or epilepsy and the temporal relationship among those events remains unknown.

An experimental model of fluorothyl-induced recurrent neocortical neonatal seizures was developed in order to overcome limitations in interpreting data from animal models to human newborns, in whom seizures present a neocortical rather than hippocampal origin (Mizrahi, 1999). The author assessed an enhanced long-term excitability in pyramidal cells of the somatosensory cortex involving a N-methyl-D-aspartate (NMDA) receptor-dependent effect on synaptic transmissions (Mizrahi, 1999).

An interesting event is that whereas seizures in the neonatal period reduced the threshold to proconvulsant molecules later in life, these animals did not develop spontaneous epileptic events. This suggests the hypothesis that spontaneous seizures might not have developed in the absence of a second hit (Spagnoli et al., 2015).

Moreover, epilepsy risk after febrile seizures has already been described to be increased in some groups of patients: children with Apgar score less than 7 at 5 mins, with cerebral palsy, a birth weight of less than 2,500 g, or a gestational age under 37 weeks PMA (Vestergaard et al., 2007). Moreover, it has been suggested that a family history of seizures, a pre-existing brain damage, and early environmental factors may be responsible for an increased susceptibility to seizure-induced brain damage (Berkovic and Scheffer, 1998).

## Pro-inflammatory Molecules Involved in CNS Inflammation

Research into the neurologic inflammation have shown that it can both, precipitate and perpetuate seizures (Wilson and Task Force Members, 2015), and the pathogenic inflammatory process can be either of peripheral, or central origin. Peripheral inflammation potentiates epileptic discharges *via* changes in ion and glutamate homeostasis, and also *via* migration of pro-inflammatory molecules from peripheral inflammatory focus to the BBB. The importance of inflammatory mediators in epilepsy has been further confirmed by examination of surgical specimen from patients undergoing surgery for refractory epilepsy. The histopathological studies suggested that persistent inflammation plays a key role in the pathogenesis of epilepsy, and the refractoriness could be a result of a subsequent brain insult (Walker et al., 2016).

Patients with focal drug-resistant epilepsy have been found to exhibit disequilibrium in the IL-1β/IL-1Ra ratio (Maroso et al., 2011a), in favor of an increase of the pro-inflammatory IL-1β. The latter is considered a mediator of brain inflammation, counteracted by the anti-inflammatory receptor antagonist (IL-1Ra; Vitaliti et al., 2014). Studies in animal models have shown that pharmacologic blockade of IL-1β biosynthesis significantly decreases seizures frequency by targeting the IL-1 converting enzyme responsible for the production of the bioactive form of this interleukin (Lehtimäki et al., 2010; Maroso et al., 2011a). The authors described a pro-inflammatory cytokine profile, according which an increase in IL-6 levels and an altered IL-1β/IL-1Ra ratio may indicate the patients in whom persistent inflammation leads to alterations of neuronal excitability (Lehtimäki et al., 2010; Vitaliti et al., 2014). These findings have been further supported by human studies in patients with epilepsy, analyzing levels of pro-inflammatory cytokines in peripheral blood (Diamond et al., 2014; Wathen and Janigro, 2014). Moreover, increased serum and cerebral spinal fluid (CSF) levels of IL-1β have been associated with an increased risk of subsequent epilepsy after moderate-to-severe brain injury (Diamond et al., 2014).

Another pro-inflammatory molecule involved in the CNS inflammation has been identified in the high-mobility group box-1 (HMGB1), involved in neuroinflammation and injury evoked by epileptogenic process (Maroso et al., 2010). Importantly, this cytokine seems to be closely involved in the generation of seizures in preclinical models of epilepsy (Maroso et al., 2010). In its physiologic form HMGB1 resides in the nucleus where it regulates transcription (Maroso et al., 2010). It is then transported to the cytoplasm in response to immune activation and acetylation of its lysine residues within the protein sequences (Maroso et al., 2010). HMGB1 is released from immune cells during injury-induced sterile inflammation and infections (Maroso et al., 2010). Moreover, necrotic cell death is responsible for the passive release of HMGB1 in its nonacetylated forms (Andersson and Tracey, 2011). The functional activity of this cytokine is then derived by posttranslational redox changes of the following cysteine residues: C23, C45, and C106. HMGB1 disulfide form, derived by a disulfide bond between C23 and C45 (Yang et al., 2012), binds and signals *via* TLR-4 and induces neuromodulatory and pro-inflammatory effects through an activation of a nuclear factor kappa-B (NF-kB; Zurolo et al., 2011; Schiraldi et al., 2012). HMGB1, in its fully reduced form, acts as chemoattractant *via* complex formation with CXCL12, binding exclusively *via* CXCR4 (Schiraldi et al., 2012). Studies on experimental models of epilepsy suggest that the acetylated, disulfide form of HMGB1 determines the detrimental effects of inflammation in epilepsy (Balosso et al., 2014). Although a pilot study in patients with focal DRE suggested that HMGB1 may be a candidate biomarker in epilepsy (Walker et al., 2016), HMGB1 is not specific to epileptic process; it’s presence has been associated with autoimmune and malignant diseases (Pilzweger and Holdenrieder, 2015).

Although pharmacologic inhibition of HMGB1 has been successful in experimental models of epilepsy (Musumeci et al., 2014). HMGB1 lacks specificity for CNS. Therefore, further studies will be needed to clarify whether peripheral or CNS inflammation play a role in seizure disorders and the therapeutic consequences of their modulation.

Literature describes the involvement of other pro-inflammatory cytokines in epileptogenesis. A study on 23 Chinese subjects with West syndrome described the presence of increased levels of serum interleukin-2 (IL-2), TNF-α, and Interferon-alpha (IFN-α) compared to matched controls (Liu et al., 2001). Increased levels of IL-1β, and IL-1β Ra, and the β-amyloid precursor were also described in specimens of patients with refractory temporal lobe epilepsy (TLE), who underwent surgery for glioneural tumors or focal cortical dysplasia (Sheng et al., 1994). In these patients, levels of pro-inflammatory cytokines directly correlated with seizures frequency (Vezzani and Baram, 2007). An overexpression of nuclear factor-kB (NF-kB) was described in hippocampus areas of neuronal loss from 18 patients with medial TLE, compared with specimens from seven hippocampi of non-epileptic patients or those with cryptogenic medial TLE without hippocampal sclerosis (*p* < 0.01; Crespel et al., 2002; Vezzani and Baram, 2007). High levels of IL-17 were instead shown in patients with type Ia, IIa, and IIb focal cortical dysplasia. Moreover, protein dosages of IL-17 and IL-17R positively correlated with seizures frequency in these patients (He et al., 2013). In this study, IL-17 and IL-17R were highly expressed in neuronal microcolumns, astrocytes, dimorphic neurons, balloon cells, and vascular endothelial cells (He et al., 2013). Finally, increased levels of IL-6 were demonstrated in 22 subjects within 24 h following tonic-clonic seizures, compared with 18 matched controls (*p* < 0.01; Peltola et al., 2002). The same group also described a serum immunologic profile for patients with refractory tonic-clonic seizures, characterized by increased levels of IL-6, low levels of IL-1Ra and low IL-1Ra:IL-1β ratio, with no evidence of corresponding increase in the production from peripheral blood mononuclear cells suggesting the origin of these cytokines to be within the brain (Lehtimäki et al., 2004, 2010).

## Hormonal and Metabolic Factors Involved in the CNS Inflammation

Among biological markers that may be considered to assess vulnerability to a given phenotype, the brain-derived neurotrophic factor (BDNF) seems to play an important role. More recent animal studies have demonstrated that all animals displaying a depression-like phenotype showed low levels of BDNF after an intense stress (social defeat) and a state of oxidative stress associated with a BDNF decrease. After a couple of weeks, the depression-like profile seemed to resolve, but half of the samples maintained low serum BDNF levels and oxidative stress (Blugeot et al., 2011). Moreover, those animals with low sustained serum BDNF levels (called by the authors “vulnerable”) showed a depression-like profile when challenged by minor stressful events. Those subjects with normal BDNF levels (called “non-vulnerable”) did not display any phenotype (Blugeot et al., 2011). Therefore, the primitive stressful event sensitized a proportion of the subjects, making them vulnerable to any subsequent stress. In case this last (minor stress) would be an epileptogenic insult (such as in case of kainic acid-induced status epilepticus), vulnerable animals showed a lower threshold for the epileptic event; these subjects developed epilepsy much faster than non-vulnerable samples, displaying severe cognitive deficits and a depression-like profile (Becker et al., 2015). Therefore, the authors speculated that serum BDNF levels might be considered predictive for the vulnerability to develop epilepsy after an insult, and for the development of co-morbidities during the chronic phase with the possibility of spontaneous seizures. Similar results were described in other studies (Liu et al., 2013).

Human studies have focused on West Syndrome as an epilepsy model. West syndrome is a rare epileptic disorder usually presenting before the age of 2, characterized by infantile spasms (IS), developmental regression and hypsarrhythmic pattern on interictal electroencephalography (EEG; Engel and International League Against Epilepsy (ILAE), 2001). The etiology could be genetic, cryptogenetic, or structural/metabolic (Berg et al., 2010). Studies demonstrated that nitric oxide metabolites and nitrates both in serum and in the CSF of IS children could differentiate symptomatic from cryptogenetic etiologies, although they could not be used for prognosis (Vanhatalo and Riikonen, 2001). The pathophysiologic mechanism of IS remains unclear. Stressful event early in life has been suggested as a possible trigger (Baram et al., 1992). It is already known that the corticotropin-releasing factor (CRF) is a strong convulsive neuropeptide during early brain development; this factor acutely stimulates hypopituitary adrenocorticotropic hormone (ACTH) secretion. When CRF is chronically secreted in brain, it desensitizes CRF receptors with a consequential reduction of ACTH release (Brunson et al., 2001). Moreover; when stressful events occur repetitively, the synthesis of insulin-like growth factors is altered (IGF-1) since IGF needs a continuous influx of steroids. The reduced IGF-1 levels lead to a synaptic impairment, manifesting as alteration of cognitive functions and potentially contribute to the development of epileptic encephalopathy (Pitkänen, 2002). The authors suggested three phases of epileptic process: initial insult, latency period (epileptogenesis), and recurrence of seizures (Pitkänen, 2002). An early brain damaging insult occurred during the perinatal period is usually followed some months later by IS. Based on the above, the latent process between brain injury and onset of spasms may be a target for therapeutic intervention.

ACTH, the immunosuppressor, is included in the first-line treatment for IS, even though its therapeutic mechanism remains unknown. It has been proposed that ACTH, glucocorticoids, and the ketogenic diet, all affect IGF-1 levels to which growing brain is extremely sensitive to (Cheng et al., 2003; Agha and Monson, 2007). All these three treatment modalities also reduce neuroinflammation and they all have been used to treat IS (Corvin et al., 2012). Infants with symptomatic IS have been found to have markedly lower CSF IGF-1 levels and significantly lower ACTH concentrations when compared to infants with idiopathic disease (Riikonen et al., 2010). Moreover, children with symptomatic IS, often have a history of prenatal, perinatal or postnatal brain insult. Interestingly, animal studies have shown that prenatal stress can decrease CNS IGF-1 levels (Fregni and De Poli, 1954). Low levels of CSF IGF-1 have been correlated with poor response to conventional treatments, as well as an impaired cognitive development in children with a history of IS. The likely underlying reason is an impaired steroid synthesis and subsequent impaired stimulation of CNS IGF-1 secretion, which is essential for the survival of synapses. For these reasons, IGF-1 has been proposed as a biomarker for the IS severity. Considering that patients with low CSF IGF-1 do not respond well to therapy, an association between IGF-1 levels and subsequent degree of developmental and cognitive impairment has been proposed (Riikonen et al., 2010). In children with IS, IGF-1 levels in CSF at the time of diagnosis appear to be a biomarker of response to therapy, progression of epilepsy, and of later cognitive outcome (Riikonen et al., 2010).

Nevertheless, while some studies have demonstrated the relationship between hormones and neurotrophic factors in the disease pathogenesis, a number of additional proteins have been identified in the serum of patients with epilepsy, such as heat shock proteins 7,076 and copeptin 77; but the role of those is yet unclear (Riikonen et al., 2010).

## The Role of the Blood-Brain Barrier in the Pathogenesis of Epileptic Encephalopathy

Since the 1950s, *in vivo* studies in animal models have demonstrated the presence of a BBB dysfunction following prolonged seizures (Hu et al., 2000).

Studies supporting evidence for a role of inflammation in epilepsy are based on the analysis of the levels of the aforementioned pro-inflammatory cytokines. For example, it has been demonstrated that IL-1β, its receptor (IL-1R) and the antagonist of its receptor (IL-1Ra) are up-regulated in rodent epilepsy model after electrically and chemically induced seizures (Vezzani et al., 2008). In the same study, an increased expression of the IL-1β in glial cells continued for up to 60 days after experimentally induced status epilepticus (De Simoni et al., 2000). Nevertheless, overexpression is not the only pathogenic immune mechanism subtending prolonged seizures, as both, IL-1β and IL-1Ra can modulate susceptibility to seizures. Animal studies provided further evidence that IL-1β administered intrathecally exacerbates seizures induced by kainic acid and bicuculine (Vezzani et al., 1999), and decreased seizure threshold in animal models of febrile convulsions (Dubé et al., 2005; Heida and Pittman, 2005).

The pro-convulsive action of IL-1β seems to be associated with an IL-1R1-dependent activation of neuronal sphingomyelinase and Src kinases, and a consequent phosphorylation of the NR2B subunit of the NMDA receptor. This process leads to NMDA stabilization at the cell surface, enhanced NMDA-mediated calcium conductance, and increase of the glutamatergic neurotransmission, with consequent excitotoxicity (Viviani et al., 2003; Balosso et al., 2008).

Other studies suggested a different pathogenic mechanism of IL-1β, leading to a reduction in glutamate uptake within astrocytes (Bezzi et al., 2001), an increased glutamate release from glial cells *via* enhanced TNF- α secretion (Viviani et al., 2007), and a subsequent channel modulation (Kulkarni and Dhir, 2009).

As mentioned above, other pro-inflammatory cytokines have also been proposed to contribute to inflammatory process associated with seizures and epileptogenesis, such as TGF-β, TNF-α, and Cyclo-oxygenase 2, all occurring in the processes of the BBB disruption (Riazi et al., 2010). Recent studies have identified the BBB disruption, specifically loss of the BBB integrity and a subsequent alteration of leukocyte-endothelial permeability (Ransohoff, 2009), as a major step in CNS involvement in the systemic inflammation. This phenomenon was described as “immunological barrier to electrical storms” (Theoharides, 1990). This theory suggests that mast cells can be considered “the immune gate of the brain” (Esposito et al., 2002). Mast cells are distributed in high number within the human BBB, and in the hypothalamus (Esposito et al., 2002). After any critical stress (oxidative and/or inflammatory insult), mast cells are critically activated, with consequent BBB disruption (Theoharides and Konstantinidou, 2007). In this context, stress hormones, such as the CRF can activate mast cells in the BBB, causing its disruption (Rehni et al., 2010). Other studies described the involvement of Histamine-1 receptor in the BBB disruption, secreted by mast cells activation (Suemitsu et al., 2010).

Mast-cells-mediated local neuronal inflammation can represent the initial focus of an epileptogenic area after BBB disruption. This process seems to worsen through activation of Fc-gamma receptors (FcgRI) sited on the surface of neuronal cells, contributing to cell death after injection of kainic acid (van der Kleij et al., 2010). Therefore, authors speculated that systemic inflammatory triggers might directly affect neurons through activation of these specific receptors (Fiuza et al., 2003; van der Kleij et al., 2010).

All the pathogenic mechanism above described are not mutually exclusive: HMGB1 has been demonstrated to enhance the expression of IL-1β *via* toll-like receptors (TLRs) interaction, and to increase the expression of vascular cell adhesion proteins in the cerebrovascular endothelium (Fiuza et al., 2003); IL-1-β promotes the nucleus to cytoplasm transfer of HMGB1 (Friedman and Dingledine, 2011), while several inflammatory molecules are responsible for the BBB disruption, perpetuating the immune-mediated pathogenesis (Marchi et al., 2012), and ultimately impacting on excitatory transmission and glutamate-mediated excitotoxicity (Marchi et al., 2012).

Considering the key role of the BBB in epilepsy, biomarkers associated with its disruption have been a focus of a great interest (Hoffmann et al., 2011). Three approaches to assess BBB function have been proposed, all similar to cerebrovascular integrity studies in other neurologic diseases. The first proposal focuses on the ratio of serum and CSF albumin. The rationale is based on the fact that the BBB is a vascular barrier, which protects the brain from harmful substances from the bloodstream; and at the same time, it supplies the brain with nutrients required for proper function. BBB then regulates the trafficking of cells and molecules from the blood into the brain, segregating impermeant macromolecules (>500 Da) in the brain and blood compartments. Albumin is 10 times more concentrated in the bloodstream which leads to stable blood-to-CSF ratio with intact BBB (Hoffmann et al., 2011).

The second approach focuses on contrast-enhanced magnetic resonance imaging (MRI), in which the “ratio” between brain and blood is measured topographically, and the distribution of a marker injected systemically is visualized in the brain. The absence of extravasation indicates that the BBB is intact; the opposite can be quantified and compared across hemispheres (Hoffmann et al., 2011). This approach is effective to detect physical BBB disruption; unfortunately, it was shown inefficient for detecting alteration of molecular trafficking and permeability within the barrier.

The last approach is based on the detection of blood markers, such as specific proteins produced by the BBB, which are “segregated” in the CNS when the barrier is intact (Bezzi et al., 2001). When the barrier is disrupted, these proteins that are usually present in high concentrations in the CNS are free to diffuse into the blood following a “concentration gradient.”

Taking all this together, an ideal peripheral marker of clinical significance should have the following characteristics: (1) molecules present in brain and CSF in higher concentrations in the brain parenchyma than in plasma; (2) present at low or undetectable levels in serum of normal subjects; (3) available for extravasation in case of BBB opening; and (4) released by brain cells in response to any brain damage (e.g., during reactive gliosis; Hoffmann et al., 2011). Several proteins, such as neuron-specific enolase (NSE), S100B, and glial fibrillary acidic protein (GFAP) have been identified as having these features (Hoffmann et al., 2011).

Within the above-mentioned approaches, current imaging techniques lack the high resolution necessary to visualize the BBB, even if contrast agents have been used to measure its integrity. Functional assessment of the BBB status by calculation of the CSF-serum albumin quotient (QA) associated with contrast-enhanced MRI is widely described as the gold standards for BBB permeability (Blyth et al., 2009). Recent literature data have reported that serum S100B correlates with QA (Welch et al., 2015), allowing indirect measurement of CSF proteins without a spinal tap. In this regard, data from the literature describe equivalence between S100B negative predictive value and contrast brain neuroimaging (Welch et al., 2015). Serum S100B has been also studied as a marker of epileptogenesis after traumatic brain injury (TBI) and it has been shown to be the best marker for concussion (Babcock et al., 2006). Moreover, S100B has emerged as a candidate peripheral biomarker of BBB permeability, as increased levels of S100B have been shown to reflect the presence of damages in the BBB, thus confirming or ruling out a brain injury (Welch et al., 2015). Since serious events involving CNS are associated with an increased risk for seizures, perhaps S100B might be a useful marker to identify individuals at high risk of posttraumatic CNS complications (Welch et al., 2015).

Taken together these data show how inflammation may represent a new target for pharmacological interventions in DRE, by interfering and modifying the inflammatory process subtending recurrent seizures.

## Toll-Like Receptor Activation in Epileptic Encephalopathy

As above described, extensive literature data described the involvement of pro-inflammatory cytokines in epileptogenesis. Nevertheless, it is useful to describe the origin of these molecules, both for diagnosis and therapeutic purposes. Pro-inflammatory cytokines are produced through activation of TLRs by endogenous and exogenous ligands (Alexopoulou et al., 2001; Iliev et al., 2004; Jack et al., 2005).

Experimental studies in animal models have shown that LPS (a TLR4 ligand) intracerebral injection is associated with a decreased seizure threshold. As a consequence, LPS-treated rats, during a period of postnatal development, showed a decreased seizure threshold after stimulation with kainic acid, lithium-pilocarpine, or pentylenetetra-zole (molecules with a pro-convulsing effect; Alexopoulou et al., 2001; Iliev et al., 2004; Jack et al., 2005). In these rats increased levels of pro-inflammatory cytokines and neurodegeneration were described (Farina et al., 2005). Moreover, murine models of electrophysiological studies showed increased epileptiform discharges in response to cortical application of LPS; this cortical activation could be inhibited by application of IL-1Ra (Farina et al., 2007).

Further studies on the HMGB1, which binds TLR4, showed that the use of HMGB1 antagonist BoxA (a HMGB1 fragment with receptor antagonist activity), as well as TLR4 antagonists were associated with a reduced frequency of kainate- and bicuculline-triggered seizures (Bsibsi et al., 2002). The same authors showed that TLR-4 knockout mice were resistant to any epileptic stimuli (Bsibsi et al., 2002). In addition, studies on surgical specimens from patients affected by ganglogliomas, focal cortical dysplasia, and tuberous sclerosis demonstrated the presence of increased expression of TLR2- and TLR4- mRNAs, RAGE mRNA and HMGB1, when the brain tissue from epileptic patients was compared to controls (Olson and Miller, 2004). The authors found that TLR2 was expressed by activated microglia, TLR4 by neurons and astrocytes, RAGE by neurons and glial cells, and HMGB1 in the nuclei of neurons and glial cells in normal brain specimens, while in pathological specimens HMGB1 was expressed in the cytoplasm of activated microglia and astrocytes (Olson and Miller, 2004). In those pathological specimens, IL-1β was responsible for the nucleus-to-cytoplasm translocation of HMGB1 (Olson and Miller, 2004).

Studies showing an increased expression of HMGB1 and TLR4 in tissue specimens from patients with epileptic encephalopathy provide further evidence for their fundamental role in epileptogenesis. HMGB1 acts by activation and interaction of NMDA receptors (Kigerl et al., 2007). The release of HMGB-1 and IL-1β from astrocytes is responsible for the activation of the IL-1receptor (IL-1R)/TLR, with consequent initiation of the TLR signaling and final activation of NF-kB. As a result, one of the NMDA receptor subunits is phosphorylated leading to calcium influx into neurons and increasing cellular excitability (Kigerl et al., 2007). Moreover, IL-1β inhibits glutamate reuptake by astrocytes and induces glutamate release by glia. These processes lead to a glutamate imbalance that further increases neuronal hyperexcitability (Kigerl et al., 2007). Finally, literature data showed that TLR3 plays a crucial role in epileptogenesis, as in febrile seizures secondary to a viral infection; as TLR3 interacts with viral pathogen-associated molecular patterns (PAMPs; Maroso et al., 2011b). Intracerebrovascular injection of poly I:C (TLR3 ligand) in 14-day old murine models caused fever and raised IL-1β levels in their brains. These rats were also more susceptible to pentylenetetrazol- and lithium-pilocarpine- induced seizures, showing, later in life, increased levels of NMDA and AMPA receptors subunits, as a consequence of increased mRNA expression (Maroso et al., 2011b).

The sustained production of IL-1β by activated astrocytes suggests a crucial role for these cells in epileptogenesis. The involvement of the IL-1β/IL-1β R axis seems to be strictly associated with the secretion of the intracellular protein MyD88, which interacts with TLR4 during pathogen recognition (Maroso et al., 2010). Nevertheless, the role of this protein remains unclear as only few studies investigated its pathophysiology in epileptogenesis. In addition, TLR-signaling pathways are able to recognize molecules secreted by damaged tissues: the damage-associated molecule patterns (DAMPs), which include HMGB1 (Maroso et al., 2010). Maroso et al. (2010) used chemical epilepsy models, by the administration of kainic acid and bicuculline, to show the nature of the HMGB1-TLR4 interaction, their intracellular signaling pathway, and their role in epilepsy genesis and recurrence. In their study, a hippocampal increase of TLR4 and HMGB1 expression in neurons, microglia, and astrocytes were described in murine models following intrahippocampal injection of kainic acid and bicuculline. The same result was found in human hippocampal tissues from intractable TLE patients (Maroso et al., 2010). The administration of recombinant HMGB1 to the studied murine pre-treated with kainic acid had a proconvulsant effect that was not observed in mice defective for TLR4 signaling. Therefore, the authors suggested that HMGB1-TLR4 signaling is strictly involved in epileptogenesis. Nevertheless, it was not possible to describe the origin of HMGB1 secretion and the triggers involved in HMGB1 and TLR4 expression in neurons and glia during seizures (Maroso et al., 2010; Matin et al., 2015). However, the authors were able to show that ictal activity itself may determine the rate of synthesis and release of HMGB1 from murine glial cells. Furthermore, HMGB1-TLR4 antagonists decreased the number and duration of seizures, increasing the onset latency period in the bicuculline-induced non-lesional models. The study also showed the extent to which HMGB-1-TLR4 signaling pathway influences chronic epilepsy. It suggested that HMGB1 and TLR4 receptor antagonists were able to block both acute seizures secondary to bicuculline and kainic acid injection and recurrence of seizures in the C57BL/6 mouse model of chronic epilepsy (Maroso et al., 2010; Matin et al., 2015). These data suggest that neuronal epileptic activity is sufficient to facilitate HMGB1 synthesis and subsequent release from cells (Maroso et al., 2010; Matin et al., 2015). However, these studies have not given evidence for continuous HMGB1 synthesis and activity, and they also did not clarify the role of the IL-1β in the process. Overall the role of HMGB1-TLR4 signaling in the genesis of epilepsy still remains to be clarified. The signaling pathway, however; may interact with only one branch of the IL-1β pathway acting with a distinct pathophysiology. Alternatively, HMGB1 may control the IL-1β pathway through primary regulation of IL-1β itself or *via* modifications of intracellular signaling components. Reduced cell death and the presence of common signaling molecules for the HMGB1 and IL-1β pro-convulsive actions, perhaps suggest that pharmacologic interventions along this pathways may lead to more effective treatments for current DRE, particularly in pediatric age population (Szczesny et al., 2014).

## Conclusion

Epileptic encephalopathy represents a treatment challenge for neurologists, pediatricians and neonatologists. Identification of biological markers as an expression of the involvement of the immune system in the disease etiology is fundamental to both, determination of the prognosis following the first seizure as well as the likelihood of drug-resistance. Unfortunately, so far many of the above-mentioned biomarkers have not been seizure or epilepsy specific, as they have also been implicated in the pathophysiology of other neurological diseases. Further research will have to address the disease-individuality and identify panels of molecules that discriminate epilepsies from other neurological diseases and perhaps distinguish between different etiologies of epilepsy. Early identification of biological markers of aberrant inflammation could potentially further help to classify the type of epilepsy, even identify etiological diagnosis of disease early, before the inflammation worsens seizure control or contributes to status epilepticus.

Currently, the markers of BBB integrity have been proven to be useful in determining the sequelae in a variety of neurologic diseases. Nevertheless, how these markers may predict the diagnosis and prognosis of epileptic disorders is currently focus of intensive research. From experimental work we have learnt that certain biomarkers have been shown to be involved in the BBB disruption and secondary epileptogenesis. Further studies focused on their clinical significance are needed to identify clinical biomarkers with diagnostic and prognostic value in epileptogenesis.

## Author Contributions

GV, GC, and RF drafted the article, provided literature data, wrote this manuscript, and gave final consent for publication. PP, SM, and MS provided literature data, revised the final version of this manuscript, and gave final consent for publication.

## Conflict of Interest Statement

The authors declare that the research was conducted in the absence of any commercial or financial relationships that could be construed as a potential conflict of interest.

## References

[B1] Agha A. Monson J. P. (2007). Modulation of glucocorticoid metabolism by the growth hormone - IGF-1 axis. Clin. Endocrinol. 66, 459–465. 10.1111/j.1365-2265.2007.02763.x 17371460

[B2] Alexopoulou L. Holt A. C. Medzhitov R. Flavell R. A. (2001). Recognition of double-stranded RNA and activation of NF-kappaB by toll-like receptor 3. Nature 413, 732–738. 10.1038/35099560 11607032

[B3] Andersson U. Tracey K. J. (2011). HMGB1 is a therapeutic target for sterile inflammation and infection. Annu. Rev. Immunol. 29, 139–162. 10.1146/annurev-immunol-030409-101323 21219181 PMC4536551

[B4] Babcock A. A. Wirenfeldt M. Holm T. Nielsen H. H. Dissing-Olesen L. Toft-Hansen H.. (2006). Toll-like receptor 2 signaling in response to brain injury: an innate bridge to neuroinflammation. J. Neurosci. 26, 12826–12837. 10.1523/jneurosci.4937-05.2006 17151286 PMC6674840

[B5] Balosso S. Maroso M. Sanchez-Alavez M. Ravizza T. Frasca A. Bartfai T.. (2008). A novel non-transcriptional pathway mediates the proconvulsive effects of interleukin-1β. Brain 131, 3256–3265. 10.1093/brain/awn271 18952671 PMC2724908

[B6] Baram T. Z. (2012). The brain, seizures and epilepsy throughout life; understanding a moving target. Epilepsy Curr. 12, 7–12. 10.5698/1535-7511-12.4s.7 23476117 PMC3588148

[B7] Baram T. Z. Mitchell W. G. Snead O. C. III. Horton E. J. Saito M. (1992). Brain-adrenal axis hormones are altered in the CSF of infants with massive infantile spasms. Neurology 42, 1171–1175. 10.1212/wnl.42.6.1171 1318521 PMC3139472

[B8] Becker C. Bouvier E. Ghestem A. Siyoucef S. Clasverie D. Camus F.. (2015). Predicting and treating stressinduced vulnerability to epilepsy and depression. Ann. Neurol. 78, 128–136. 10.1002/ana.24414 25869354

[B9] Berg A. T. Berkovic S. F. Brodie M. J. Buchhalter J. Cross J. H. van Emde Boas W.. (2010). Revised terminology and concepts for organization of seizures and epilepsies: report of the ILAE commission on classification and terminology, 2005–2009. Epilepsia 51, 676–685. 10.1111/j.1528-1167.2010.02522.x 20196795

[B10] Berkovic S. F. Scheffer I. E. (1998). Febrile seizures: genetics and relationship to other epilepsy syndromes. Curr. Opin. Neurol. 11, 129–134. 10.1097/00019052-199804000-00009 9551293

[B11] Bezzi P. Domercq M. Brambilla L. Galli R. Schols D. De Clercq E.. (2001). CXCR4-activated astrocyte glutamate release via TNFα: amplification by microglia triggers neurotoxicity. Nat. Neurosci. 4, 702–710. 10.1038/89490 11426226

[B12] Blugeot A. Rivat C. Bouvier E. Molet J. Mouchard A. Zeau B.. (2011). Vulnerability to depression: from brain neuroplasticity to identification of biomarkers. J. Neurosci. 31, 12889–12899. 10.1523/jneurosci.1309-11.2011 21900567 PMC6623387

[B13] Blyth B. J. Farhavar A. Gee C. Molet J. Mouchard A. Zeau B.. (2009). Validation of serum markers for blood-brain barrier disruption in traumatic brain injury. J. Neurotrauma 26, 1497–1507. 10.1089/neu.2008-0738 19257803 PMC2822805

[B14] Brunson K. L. Khan N. Eghbal-Ahmadi M. Baram T. Z. (2001). Corticotropin (ACTH) acts directly on amygdala neurons to down-regulate corticotropinreleasing hormone gene expression. Ann. Neurol. 49, 304–312. 10.1002/ana.66 11261504 PMC2849730

[B15] Bsibsi M. Ravid R. Gveric D. van Noort J. M. (2002). Broad expression of Toll-like receptors in the human central nervous system. J. Neuropathol. Exp. Neurol. 61, 1013–1021. 10.1093/jnen/61.11.1013 12430718

[B16] Cheng C. M. Kelley B. Wang J. Strauss D. Eagles D. A. Bondy C. A. (2003). A ketogenic diet increases brain insulin-like growth factor receptor and glucose transporter gene expression. Endocrinology 144, 2676–2682. 10.1210/en.2002-0057 12746332

[B17] Corvin A. P. Molinos I. Little G. Donohoe G. Gill M. Morris D. W.. (2012). Insulin-like growth factor 1 (IGF1) and its active peptide (1–3) IGF1 enhance the expression of synaptic markers in neuronal circuits through different cellular mechanisms. Neurosci. Lett. 520, 51–56. 10.1016/j.neulet.2012.05.029 22609570

[B18] Crespel A. Coubes P. Rousset M. C. Brana C. Rougier A. Rondouin G.. (2002). Inflammatory reactions in human medial temporal lobe epilepsy with hippocampal sclerosis. Brain Res. 952, 159–169. 10.1016/s0006-8993(02)03050-0 12376176

[B19] De Simoni M. G. Perego C. Ravizza T. Moneta D. Conti M. Marchesi F.. (2000). Inflammatory cytokines and related genes are induced in the rat hippocampus by limbic status epilepticus. Eur. J. Neurosci. 12, 2623–2633. 10.1046/j.1460-9568.2000.00140.x 10947836

[B20] Diamond M. L. Ritter A. C. Failla M. D. Boles J. A. Conley Y. P. Kochanek P. M.. (2014). IL-1β associations with posttraumatic epilepsy development: a genetics and biomarker cohort study. Epilepsia 55, 1109–1119. 10.1111/epi.12628 24754437 PMC4107119

[B21] Dubé C. Vezzani A. Behrens M. Bartfai T. Baram T. Z. (2005). Interleukin-1β contributes to the generation of experimental febrile seizures. Ann. Neurol. 57, 152–155. 10.1002/ana.20358 15622539 PMC2909879

[B22] Ellenberg J. H. Hirtz D. G. Nelson K. B. (1984). Age at onset of seizures in young children. Ann. Neurol. 15, 127–134. 10.1002/ana.410150204 6703653

[B23] Engel and International League Against Epilepsy (ILAE) J. Jr. International League Against Epilepsy (ILAE). (2001). A proposed diagnostic scheme for people with epileptic seizures and with epilepsy: report of the ILAE Task Force on classification and terminology. Epilepsia 42, 796–803. 10.1046/j.1528-1157.2001.10401.x 11422340

[B24] Esposito P. Chandler N. Kandere K. Basu S. Jacobson S. Connolly R.. (2002). Corticotropin-releasing hormone and brain mast cells regulate blood-brain-barrier permeability induced by acute stress. J. Pharmacol. Exp. Ther. 303, 1061–1066. 10.1124/jpet.102.038497 12438528

[B25] Farina C. Aloisi F. Meinl E. (2007). Astrocytes are active play- ers in cerebral innate immunity. Trends Immunol. 28, 138–145. 10.1016/j.it.2007.01.005 17276138

[B26] Farina C. Krumbholz M. Giese T. Hartmann G. Aloisi F. Meinl E. (2005). Preferential expression and function of Toll-like receptor 3 in human astrocytes. J. Neuroimmunol. 159, 12–19. 10.1016/j.jneuroim.2004.09.009 15652398

[B27] Fiuza C. Bustin M. Talwar S. Tropea M. Gerstenberger E. Shelhamer J. H.. (2003). Inflammation-promoting activity of HMGB1 on human microvascular endothelial cells. Blood 101, 2652–2660. 10.1182/blood-2002-05-1300 12456506

[B28] Fregni R. De Poli A. (1954). Convulsive state produced by various types of shock; conduct of three barriers (blood-aqueous, blood-labyrinthine fluids and blood-liquor [spinal fluid]) with reference to some convulsive states. AMA Arch. Otolaryngol. 60, 149–153. 10.1001/archotol.1954.00720010155004 13180062

[B29] Friedman A. Dingledine R. (2011). Molecular cascades that mediate the influence of inflammation on epilepsy. Epilepsia 52, 33–39. 10.1111/j.1528-1167.2011.03034.x 21542844 PMC3638118

[B30] He J. J. Li S. Shu H. F. Yu S. X. Liu S. Y. Yin Q.. (2013). The interleukin 17 system in cortical lesions in focal cortical dysplasias. J. Neuropathol. Exp. Neurol. 72, 152–163. 10.1097/NEN.0b013e318281262e 23334598

[B31] Heida J. G. Pittman Q. J. (2005). Causal links between brain cytokines and experimental febrile convulsions in the rat. Epilepsia 46, 1906–1913. 10.1111/j.1528-1167.2005.00294.x 16393156

[B32] Herrgård E. A. Karvonen M. Luoma L. Saavalainen P. Määttä S. Laukkanen E.. (2006). Increased number of febrile seizures in children born very preterm: relation of neonatal, febrile and epileptic seizures and neurological dys- function to seizure outcome at 16 years of age. Seizure 15, 590–597. 10.1016/j.seizure.2006.08.004 16990025

[B33] Hoffmann A. Bredno J. Wendland M. F. Derugin N. Hom J. Schuster T.. (2011). Validation of *in vivo* magnetic resonance imaging blood-brain barrier permeability measurements by comparison with gold standard histology. Stroke 42, 2054–2060. 10.1161/strokeaha.110.597997 21636816 PMC3921072

[B34] Hoffmann A. F. Zhao Q. Holmes G. L. (2004). Cognitive impairment following status epilepticus and recurrent seizures during early development: support for the “two-hit hypothesis”. Epilepsy Behav. 5, 873–877. 10.1016/j.yebeh.2004.09.005 15582835

[B35] Hu S. Sheng W. S. Ehrlich L. C. Peterson P. K. Chao C. C. (2000). Cytokine effects on glutamate uptake by human astrocytes. Neuroimmunomodulation 7, 153–159. 10.1159/000026433 10754403

[B36] Hulkkonen J. Koskikallio E. Rainesalo S. Keränen T. Hurme M. Peltola J. (2004). The balance of inhibitory and excitatory cytokines is differently regulated *in vivo* and *in vitro* among therapy-resistant epilepsy patients. Epilepsy Res. 59, 199–205. 10.1016/j.eplepsyres.2004.04.007 15246121

[B37] Iliev A. I. Stringaris A. K. Nau R. Neumann H. (2004). Neuronal injury mediated via stimulation of microglial toll- like receptor-9 (TLR9). FASEB J. 18, 412–414. 10.1096/fj.03-0670fje 14688201

[B38] Jack C. S. Arbour N. Manusow J. Montgrain V. Blain M. McCrea E.. (2005). TLR signaling tailors innate immune responses in human microglia and astrocytes. J. Immunol. 175, 4320–4330. 10.4049/jimmunol.175.7.4320 16177072

[B39] Kigerl K. A. Lai W. Rivest S. Hart R. P. Satoskar A. R. Popovich P. G. (2007). Toll-like receptor (TLR)-2 and TLR-4 regulate inflammation, gliosis and myelin sparing after spinal cord injury. J. Neurochem. 102, 37–50. 10.1111/j.1471-4159.2007.04524.x 17403033

[B40] Koh S. Storey T. W. Santos T. C. Mian A. Y. Cole A. J. (1999). Early-life seizures in rats increase susceptibility to seizure-induced brain injury in adulthood. Neurology 53, 915–921. 10.1212/wnl.53.5.915 10496246

[B41] Kulkarni S. K. Dhir A. (2009). Cyclooxygenase in epilepsy: from perception to application. Drugs Today 45, 135–154. 10.1358/dot.2009.45.2.1322481 19343233

[B42] Lehtimäki K. A. Keränen T. Huhtala H. Hurme M. Ollikainen J. Honkaniemi J.. (2004). Regulation of IL-6 system in cerebrospinal fluid and serum compartments by seizures: the effect of seizure type and duration. J. Neuroimmunol. 152, 121–125. 10.1016/j.jneuroim.2004.01.024 15223244

[B43] Lehtimäki K. A. Keränen T. Palmio J. Peltola J. (2010). Levels of IL-1β and IL-1ra in cerebrospinal fluid of human patients after single and prolonged seizures. Neuroimmunomodulation 17, 19–22. 10.1159/000243081 19816053

[B44] Liu G. Gu B. He X. P. Wackerle H. D. Rodriguiz R. M. Wetsel W. C.. (2013). Transient inhibition of TrkB kinase after status epilepticus prevents development of temporal lobe epilepsy. Neuron 79, 31–38. 10.1016/j.neuron.2013.04.027 23790754 PMC3744583

[B45] Liu Z. S. Wang Q. W. Wang F. L. Yang L. Z. (2001). Serum cytokine levels are altered in patients with West syndrome. Brain Dev. 23, 548–551. 10.1016/s0387-7604(01)00313-8 11701253

[B46] Mao L. Y. Ding J. Peng W. F. Ma Y. Zhang Y. H. Fan W.. (2013). Interictal interleukin-17A levels are elevated and correlate with seizure severity of epilepsy patients. Epilepsia 54, e142–e145. 10.1111/epi.12337 23944193

[B47] Marchi N. Granata T. Freri E. Ciusani E. Ragona F. Puvenna V.. (2011). Efficacy of anti-inflammatory therapy in a model of acute seizures and in a population of pediatric drug-resistant epileptics. PLoS One 6:e18200. 10.1371/journal.pone.0018200 21464890 PMC3065475

[B48] Marchi N. Granata T. Ghosh C. Janigro D. (2012). Blood-brain barrier dysfunction and epilepsy: pathophysiologic role and therapeutic approaches. Epilepsia 53, 1877–1886. 10.1111/j.1528-1167.2012.03637.x 22905812 PMC4842020

[B50] Maroso M. Balosso S. Ravizza T. Iori V. Wright C. I. French J.. (2011a). Interleukin-1β biosynthesis inhibition reduces acute seizures and drug-resistant chronic epileptic activity in mice. Neurotherapeutics 8, 304–315. 10.1007/s13311-011-0039-z 21431948 PMC3101825

[B49] Maroso M. Balosso S. Ravizza T. Liu J. Bianchi M. Vezzani A. (2011b). Interleukin-1 type 1 receptor/Toll-like receptor signalling in epilepsy: the importance of IL-1beta and high-mobility group box 1. J. Internal. Med. 270, 319–326. 10.1111/j.1365-2796.2011.02431.x 21793950

[B51] Maroso M. Balosso S. Ravizza T. Liu J. Aronica E. Iyer A. M.. (2010). Toll-like receptor 4 and high- mobility group box-1 are involved in ictogenesis and can be targeted to reduce seizures. Nat. Med. 16, 413–419. 10.1038/nm.2127 20348922

[B52] Matin N. Tabatabaie O. Falsaperla R. Lubrano R. Pavone P. Mahmoof F.. (2015). Epilepsy and innate immune system: a possible immunogenic predisposition and related therapeutic implications. Hum. Vaccin. Immunother. 11, 2021–2029. 10.1080/21645515.2015.1034921 26260962 PMC4635700

[B53] Mizrahi E. M. (1999). Acute and chronic effects of seizures in the developing brain: lessons from clinical experience. Epilepsia 40, S42–S50. 10.1111/j.1528-1157.1999.tb00878.x 10421560

[B54] Musumeci D. Roviello G. N. Montesarchio D. (2014). An overview on HMGB1 inhibitors as potential therapeutic agents in HMGB1-related pathologies. Pharmacol. Ther. 141, 347–357. 10.1016/j.pharmthera.2013.11.001 24220159

[B55] Olson J. K. Miller S. D. (2004). Microglia initiate central nervous system innate and adaptive immune responses through multiple TLRs. J. Immunol. 173, 3916–3924. 10.4049/jimmunol.173.6.3916 15356140

[B56] Peltola J. Laaksonen J. Haapala A. M. Hurme M. Rainesalo S. Keränen T. (2002). Indicators of inflammation after recent tonic-clonic epileptic seizures correlate with plasma interleukin-6 levels. Seizure 11, 44–46. 10.1053/seiz.2001.0575 11888259

[B57] Pilzweger C. Holdenrieder S. (2015). Circulating HMGB1 and RAGE as clinical biomarkers in malignant and autoimmune diseases. Diagnostics 5, 219–253. 10.3390/diagnostics5020219 26854151 PMC4665591

[B58] Pisani F. Piccolo B. Cantalupo G. Copioli C. Fusco C. Pelosi A.. (2012). Neonatal seizures and postneonatal epilepsy: a 7-y follow-up study. Pediatr. Res. 72, 186–193. 10.1038/pr.2012.66 22580721

[B59] Pitkänen A. (2002). Drug-mediated neuroprotection and antiepileptogenesis: animal data. Neurology 59, S27–S33. 10.1212/wnl.59.9_suppl_5.s27 12428029

[B60] Rakhade S. N. Jensen F. E. (2009). Epileptogenesis in the immature brain: emerging mechanisms. Nat. Rev. Neurol. 5, 380–391. 10.1038/nrneurol.2009.80 19578345 PMC2822660

[B61] Ransohoff R. M. (2009). Immunology: barrier to electrical storms. Nature 457, 155–156. 10.1038/457155a 19129836

[B62] Rehni A. K. Singh T. G. Singh N. Arora S. (2010). Tramadol-induced epileptogenic effect: a possible role of opioid-dependent histamine H1 receptor activation-linked mechanism. Naunyn Schmiedebergs Arch. Pharmacol. 381, 11–19. 10.1007/s00210-009-0476-y 20012267

[B63] Riazi K. Galic M. A. Pittman Q. J. (2010). Contributions of peripheral inflammation to seizure susceptibility: cytokines and brain excitability. Epilepsy Res. 89, 34–42. 10.1016/j.eplepsyres.2009.09.004 19804959

[B64] Riikonen R. S. Jääskeläinen J. Turpeinen U. (2010). Insulin-like growth factor-1 is associated with cognitive outcome in infantile spasms. Epilepsia 51, 1283–1289. 10.1111/j.1528-1167.2009.02499.x 20163445

[B65] Roberts D. J. Goralski K. B. (2008). A critical overview of the influence of inflammation and infection on P-glycoprotein expression and activity in the brain. Expert Opin. Drug Metab. Toxicol. 4, 1245–1264. 10.1517/17425255.4.10.1245 18798696

[B66] Schiraldi M. Raucci A. Munoz L. M. Livoti E. Celona B. Venereau E.. (2012). HMGB1 promotes recruit- ment of inflammatory cells to damaged tissues by forming a complex with CXCL12 and signaling via CXCR4. J. Exp. Med. 209, 551–563. 10.1084/jem.20111739 22370717 PMC3302219

[B67] Schmid R. Tandon P. Stafstrom C. E. Holmes G. L. (1999). Effects of neonatal seizures on subsequent seizure-induced brain injury. Neurology 53, 1754–1761. 10.1212/wnl.53.8.1754 10563624

[B68] Sheng J. G. Boop F. A. Mrak R. E. Griffin W. S. T. (1994). Increased neuronal β-amyloid precursor protein expression in human temporal lobe epilepsy: association with interleukin-1 α immunoreactivity. J. Neurochem. 63, 1872–1879. 10.1046/j.1471-4159.1994.63051872.x 7931344 PMC3833617

[B69] Spagnoli C. Cilio M. R. Pavlidis E. Pisani F. (2015). Symptomatic neonatal seizures followed by febrile status epilepticus: the two-hit hypothesis for the subsequent development of epilespy. J. Child Neurol. 30, 615–618. 10.1177/0883073814533004 24810087

[B70] Suemitsu S. Watanabe M. Yokobayashi E. Usui S. Ishikawa T. Matsumoto Y.. (2010). Fcγ receptors contribute to pyramidal cell death in the mouse hippocampus following local kainic acid injection. Neuroscience 166, 819–831. 10.1016/j.neuroscience.2010.01.004 20074624

[B71] Szczesny E. Basta-Kaim A. Slusarczyk J. Trojan E. Glombik K. Regulska M.. (2014). The impact of prenatal stress on insulin-like growth factor-1 and pro-inflammatory cytokine expression in the brains of adult male rats: the possible role of suppres- sors of cytokine signaling proteins. J. Neuroimmunol. 276, 37–46. 10.1016/j.jneuroim.2014.08.001 25151093

[B72] Theoharides T. C. (1990). Mast cells: the immune gate to the brain. Life Sci. 46, 607–617. 10.1016/0024-3205(90)90129-f 2407920

[B73] Theoharides T. C. Konstantinidou A. D. (2007). Corticotropin-releasing hormone and the blood-brain-barrier. Front. Biosci. 12, 1615–1628. 10.2741/2174 17127408

[B74] van Campen J. S. Jansen F. E. de Graan P. N. Braun K. P. Joels M. (2014). Early life stress in epilepsy: a seizure precipitant and risk factor for epileptogenesis. Epilepsy Behav. 38, 160–171. 10.1016/j.yebeh.2013.09.029 24144618

[B75] van der Kleij H. Charles N. Karimi K. Mao Y. K. Foster J. Janssen L.. (2010). Evidence for neuronal expression of functional Fc (ε and γ) receptors. J. Allergy Clin. Immunol. 125, 757–760. 10.1016/j.jaci.2009.10.054 20132972 PMC2839037

[B76] Vanhatalo S. Riikonen R. (2001). Nitric oxide metabolites, nitrates and nitrites in the cerebrospinal fluid in children with west syndrome. Epilepsy Res. 46, 3–13. 10.1016/s0920-1211(00)00154-6 11395283

[B77] Vestergaard M. Pedersen C. B. Sidenius P. Olsen J. Christensen J. (2007). The long-term risk of epilepsy after febrile seizures in susceptible subgroups. Am. J. Epidemiol. 165, 911–918. 10.1093/aje/kwk086 17267419

[B8700] Vezzani A. Baram T. Z. (2007). New roles for interleukin-1 Beta in the mechanisms of epilepsy. Epilepsy Curr. 7, 45–50. 10.1111/j.1535-7511.2007.00165.x 17505552 PMC1867084

[B78] Vezzani A. Balosso S. Ravizza T. (2008). The role of cytokines in the pathophysiology of epilepsy. Brain Behav. Immun. 22, 797–803. 10.1016/j.bbi.2008.03.009 18495419

[B79] Vezzani A. Conti M. De Luigi A. Ravizza T. Moneta D. Marchesi F.. (1999). Interleukin-1β immunoreactivity and microglia are enhanced in the rat hippocampus by focal kainate application: functional evidence for enhancement of electrographic seizures. J. Neurosci. 19, 5054–5065. 10.1523/jneurosci.19-12-05054.1999 10366638 PMC6782637

[B80] Vitaliti G. Pavone P. Mahmood F. Nunnari G. Falsaperla R. (2014). Targeting inflammation as a therapeutic strategy for drug-resistant epilepsies: an update of new immune-modulating approaches. Hum. Vaccin. Immunother. 10, 868–875. 10.4161/hv.28400 24609096 PMC4896581

[B81] Viviani B. Bartesaghi S. Gardoni F. Vezzani A. Behrens M. M. Bartfai T.. (2003). Interleukin-1β enhances NMDA receptor-mediated intracellular calcium increase through activation of the Src family of kinases. J. Neurosci. 23, 8692–8700. 10.1523/jneurosci.23-25-08692.2003 14507968 PMC6740426

[B82] Viviani B. Gardoni F. Marinovich M. (2007). Cytokines and neuronal ion channels in health and disease. Int. Rev. Neurobiol. 82, 247–263. 10.1016/s0074-7742(07)82013-7 17678965

[B83] Walker L. E. Janigro D. Heinemann U. Riikonen R. Bernard C. Patel M. (2016). WONOEP appraisal: molecular and cellular biomarkers for epilepsy. Epilepsia 57, 1354–1362. 10.1111/epi.13460 27374986 PMC5515385

[B84] Wathen C. Janigro D. (2014). IL-1β associations with posttraumatic epilepsy development: a genetics and biomarker cohort study. Epilepsia 55:1313. 10.1111/epi.12693 25099305 PMC4843779

[B87000] Welch R. D. Ayaz S. I. Lewis L. M. Unden J. Chen J. Y. Mika V. H.. (2015). Ability of serum glial fibrillary acidic protein, ubiquitin C-terminal hydrolase-L1, and S100B to differentiate normal and abnormal head computed tomography findings in patients with suspected mild or moderate traumatic brain injury. J. Neurotrauma 33, 203–214. 10.1089/neu.2015.4149 26467555 PMC4722555

[B85] Wilson S. Task Force Members. (2015). In response: indications and expectations for neuropsychological assessment in routine epilepsy care: report of the ILAE neuropsychology task force, diagnostic methods commission, 2013-2017. Epilepsia 56, 1316–1317. 10.1111/epi.13055 26235735

[B86] Yang H. Lundbäck P. Ottosson L. Erlandsson-Harris H. Venereau E. Bianchi M. E.. (2012). Redox modification of cysteine residues regulate the cytokine activity of high mobility group box-1 (HMGB1). Mol. Med. 18, 250–259. 10.2119/molmed.2011.00389 22105604 PMC3324950

[B87] Zurolo E. Iyer A. Maroso M. Carbonell C. Anink J. J. Ravizza T.. (2011). Activation of the Toll-like receptor, RAGE and HMGB1 signaling in malformations of cortical development. Brain 134, 1015–1032. 10.1093/brain/awr032 21414994

